# The role of the cerebellum in sequencing and predicting social and non-social events in patients with bipolar disorder

**DOI:** 10.3389/fncel.2023.1095157

**Published:** 2023-02-15

**Authors:** Libera Siciliano, Giusy Olivito, Michela Lupo, Nicole Urbini, Andrea Gragnani, Marco Saettoni, Roberto Delle Chiaie, Maria Leggio

**Affiliations:** ^1^Department of Psychology, Sapienza University of Rome, Rome, Italy; ^2^Ataxia Laboratory, Fondazione Santa Lucia IRCCS, Rome, Italy; ^3^Servizio di Tutela della Salute Mentale e Riabilitazione dell’Età Evolutiva ASL, Rome, Italy; ^4^Scuola di Psicoterapia Cognitiva SPC, Grosseto, Italy; ^5^Associazione Psicologia Cognitiva (APC)/Scuola di Psicoterapia Cognitiva (SPC), Rome, Italy; ^6^Unità Funzionale Salute Mentale Adulti ASL Toscana Nord-Ovest Valle del Serchio, Pisa, Italy; ^7^Department of Neuroscience and Mental Health–Policlinico Umberto I Hospital, Sapienza University of Rome, Rome, Italy

**Keywords:** bipolar disorder, theory of mind, sequential processing, prediction, cerebellar atrophy, voxel-based morphometry

## Abstract

**Introduction:**

Advances in the operational mode of the cerebellum indicate a role in sequencing and predicting non-social and social events, crucial for individuals to optimize high-order functions, such as Theory of Mind (ToM). ToM deficits have been described in patients with remitted bipolar disorders (BD). The literature on BD patients’ pathophysiology reports cerebellar alterations; however, sequential abilities have never been investigated and no study has previously focused on prediction abilities, which are needed to properly interpret events and to adapt to changes.

**Methods:**

To address this gap, we compared the performance of BD patients in the euthymic phase with healthy controls using two tests that require predictive processing: a ToM test that require implicit sequential processing and a test that explicitly assesses sequential abilities in non-ToM functions. Additionally, patterns of cerebellar gray matter (GM) alterations were compared between BD patients and controls using voxel-based morphometry.

**Results:**

Impaired ToM and sequential skills were detected in BD patients, specifically when tasks required a greater predictive load. Behavioral performances might be consistent with patterns of GM reduction in cerebellar lobules Crus I-II, which are involved in advanced human functions.

**Discussion:**

These results highlight the importance of deepening the cerebellar role in sequential and prediction abilities in patients with BD.

## 1. Introduction

Over the past three decades, the cerebellum has been recognized as playing a role in a variety of cognitive and social functions as well as affective regulation (Tedesco et al., 2011; Buckner, 2013; Mariën et al., 2014; Sokolov et al., 2017; Leggio and Olivito, 2018; Stoodley and Schmahmann, 2018; Schmahmann, 2019). However, how the cerebellum peculiarly operates in such multifarious domains is still being discussed. According to the “sequence detection theory” postulated by Leggio and colleagues (Leggio et al., 2011) the cerebellum is engaged for detecting and simulating repetitive patterns of temporally and spatially organized events. Once the sequence of events has been identified, the cerebellum contributes to the implementation of internal models that allow one to make predictions about the consequences of events and to detect any discrepancies between expected and occurring events, thus making online corrections when errors occur (Ito, 2008; Leggio et al., 2011; Leggio and Molinari, 2015). Specifically, the cerebellum compares actual input and preceding stimuli, makes associations among them in space and time, and then tests potential discordances. When an incoming stimulus corresponds to the predicted one, cerebellar output is marginal. On the contrary, when a discordance or an error is revealed, the activity of the cerebellum grows, alerting specific areas of the cerebral cortex based on the type of stimuli (Ito, 2008). Accordingly, sequencing is not acknowledged as a discrete cognitive function, instead is defined as a supramodal function that subserves several functional domains ranging from sensorimotor to advanced decision-making domains (Molinari and Masciullo, 2019), supported by different brain networks. Through the cerebello-cerebral collaboration, sequences of events that have been met earlier are employed to create internal models, in turn used to make predictions. To identify sequential relationships between events, the cerebellum must understand the links between them in the space-time domain. Each event is retained and then compared with a subsequent event, matching their characteristics so that the cerebellum can place them correctly in space and time. Subsequently, due to the presence of the already established internal models, the cerebellum makes it possible to predict the succession of future events by setting the correct excitatory levels in brain areas responsible for responding adaptively to a given pattern of stimuli if it is recognized as a previously encountered model (Molinari and Masciullo, 2019). In this way, cerebellar detection and prediction of sequential events contributes to feedforward control based on anticipation, ultimately reducing the prediction error. Thus, the cerebellum represents a chief structure to be included in the “predictive brain” (Ito, 2008; Molinari and Masciullo, 2019).

Cerebellar sequential functioning has been demonstrated to contribute to information processing in the sensorimotor system (Tesche and Karhu, 2000; Doyon et al., 2002; Restuccia et al., 2007; Morgan et al., 2021) and for cognitive functions, such as visuospatial functions (Molinari et al., 1997; Leggio et al., 1999), language (Fabbro et al., 2000; Leggio et al., 2000, 2011; Tedesco et al., 2011; Lupo et al., 2021a), verbal working memory (Silveri et al., 1998; Chiricozzi et al., 2008; Leggio et al., 2011; Sheu and Desmond, 2022) and script sequences (Leggio et al., 2008; Molinari et al., 2008; Morgan et al., 2021).

More recently, the role of the cerebellum as a sequential and prediction processor has also been investigated in the social domain, with a focus on Theory of Mind (ToM) skills. In this domain, the construction of internal models reflects the correct implementation of the sequence of social actions, thus allowing people to predict one’s own and others’ behaviors and reactions, and to adjust unexpected events when violations from predicted scenarios are met to finally adjust social interaction accordingly by modulating cerebral cortex activity (Clausi et al., 2019; Heleven et al., 2019; van Overwalle et al., 2019a,b, 2022). The contribution of lobules Crus I and II has been evidenced for those functions, which is consistent with the widespread connections the posterior cerebellum has with areas in the cerebral cortex involved in high-order functions (van Overwalle and Mariën, 2016; van Overwalle et al., 2022).

In line with sequence detection theory, the cerebellum is recognized as a prediction machine that harmonizes whole-brain function by updating certain neural networks about the required responses to a given stimulus in each context (Ito, 2008; Leggio and Molinari, 2015), providing internal coherence between externally and internally produced signals (D’Angelo and Casali, 2012), all functions needed to properly interpret events and to adapt to changes. Impaired prediction abilities have been proposed as the basis of the behavioral deficits in individuals with disorders of different etiologies, such as patients with primary cerebellar damages (Rustemeier et al., 2016; Clausi et al., 2019), individuals with neurodevelopmental conditions, such as autism (Sinha et al., 2014; Siciliano and Clausi, 2020), and psychiatric disorders, such as schizophrenia (D’Angelo and Casali, 2012; Abram et al., 2022), all conditions in which cerebellar alterations have been documented (D’Mello et al., 2015; Mothersill et al., 2016; Clausi et al., 2021b).

The acknowledged role of the cerebellum in advanced and supramodal functions involving affection and cognition has widely broadened the spectrum of pathologies in which a role of the cerebellum has been hypothesized and investigated (Baldaçara et al., 2008; D’Mello and Stoodley, 2015; Lupo et al., 2019; Moreno-Rius, 2019; Siciliano et al., 2022). It is assumed that, despite the specific symptomatology, cerebellum-related cognitive and affective signs might reflect either exaggerated-hypermetric (overshoot) or diminished-hypometric (undershoot) reactions to externally or internally triggered stimuli, likely to be busted by poor cerebellar modulatory function to widespread brain networks (Schmahmann et al., 2007). In line with this and with the above-mentioned sequence detection theory, the cerebellum guarantees the proper equilibrium between processing of endogenous and exogenous stimuli and, thus, allows to choose the best corresponding responses in the environment, by modulating the activity in specific brain networks (e.g., sensory, motor, memory, attention, language, affective and social) (Schmahmann et al., 2007; D’Angelo and Casali, 2012; Leggio and Molinari, 2015).

Recently, a particular focus has been placed on cerebellar engagement in bipolar disorders (BD). BD is a debilitating and chronic psychopathology characterized by phases of depression and mania or hypomania, with varying inter-episodes of symptoms’ remission. Specific neural mechanisms involving cortical-striatal-limbic circuits are supposed to trigger the diverse mood states that characterized the two peculiar phases of BD (Spielberg et al., 2016). While diverse meta-analytic studies have currently proven the presence of structural changes in certain subcortical areas in BD, such as hippocampus, thalamus, and amygdala (Rossi et al., 2013; Hibar et al., 2016; Ding and Hu, 2021), considerable inconsistency between studies still exists about cerebellar structural changes in BD (Ding and Hu, 2021). However, the involvement of the cerebellum in this disorder is currently being investigated on the basis of clinical and scientific evidence suggesting its implication in the pathology (Mills et al., 2005; Argyropoulos et al., 2021; Lupo et al., 2021b; Olivito et al., 2022a,b). For example, the overshoot and undershoot that characterize responses to external or internal stimuli following cerebellar alterations have been associated with the states of mania and depression typical of bipolar disorder (Schmahmann et al., 2007). An imbalanced “mind-world synchronization” driven by cerebellar dysfunctional prediction ability has been hypothesized to contribute to the onset of psychotic symptoms and might be also associated with the poor mood homeostasis that leads to depressive and manic phases typical of BD (Ito, 2008; Moberget and Ivry, 2019). In support of the hypothesis of a cerebellar role in affective regulation, the occurring of both depressive and manic symptoms has recently been reported in patients with cerebellar disease of different etiology, creating a bridge between the two disorders (Clausi et al., 2019; Lupo et al., 2019). Though, the interest in deepening the contribution of the cerebellum in BD brain networks has increased lately (Argyropoulos et al., 2021; Olivito et al., 2022a), promoted by the plethora of studies that are nowadays available about the anatomical and functional connections that link the cerebellum and subcortical limbic and cortical associative areas involved in emotional and affective regulation (Stoodley and Schmahmann, 2009; Adamaszek et al., 2017; Baumann and Mattingley, 2022; Schutter, 2022). In BD, the persistence of functional connectivity changes during the euthymic phases has been reported. It has been hypothesized that they represent a vulnerability resulting from emotional brain networks (i.e., anterior limbic network) that become hypersensitive due to acute episode, in turn representing a risk for persistent affective and cognitive disturbances in BD in the euthymic phase (Strakowski et al., 2005; Syan et al., 2018; Olivito et al., 2022a). In this regard, the interest in examining the neural and behavioral mechanisms underlying the phases of symptom remission in BD has grown. In BD patients in the euthymic phase, cognitive deficits, such as visuospatial and verbal difficulties, and affective and ToM impairments have been described, strongly impacting patients’ quality of life (Martinez-Aran et al., 2007; Bora et al., 2009; Gogos et al., 2010; Osher et al., 2011; Samamé et al., 2015; de Siqueira Rotenberg et al., 2020; Olivito et al., 2022b). Furthermore, decreased volumes in the gray matter (GM) of cerebellar vermian and posterior areas have been documented (Mills et al., 2005; Lupo et al., 2021b) as well as disrupted functional and structural connectivity, which likely represents chronic impairments that persist during periods of symptom remission (Argyropoulos et al., 2021; Olivito et al., 2022a). In addition, a recent study revealed specific patterns of GM decrease in cerebellar vermis and Crus I and II both in patients with bipolar disorder type 1 and 2 in the euthymic phase, likely to explain the occurrence of ToM deficits (Olivito et al., 2022b). It must be noted that sequential abilities and prediction abilities related to social and non-social functions have never been investigated in BD.

In a previous study, we reported ToM and cerebellar alterations in BD in the euthymic phase. In the present study, we aimed to further explore these features in this population by also focusing on sequential and predictive processing, which represent the key modes of cerebellar processing. To this end, we used implicit and explicit sequential tests with or without ToM engagement, both requiring prediction processing. Specifically, we employed the Faux Pas test (Stone et al., 1998; Liverta Sempio et al., 2005), that explicitly requires to process and predict advanced social cues and implicitly needs to order social events sequentially to be well performed, and the Sequence test (Leggio et al., 2008), that allows to evaluate sequencing and predicting abilities demanding the processing of stimuli that entail behavioral, verbal, or spatial/abstract elements. Furthermore, we aimed to verify the presence of cerebellar alterations by conducting a voxel-based morphometry (VBM) analysis on MRI data of BD patients.

## 2. Materials and methods

### 2.1. Participants

Eighteen patients with BD [mean age/SD: 39/7.99; years of education/SD: 15.56/2.66; M/F: 6/12] were enrolled for this study. The present study involved BD individuals who were previously recruited for other studies from our group (Lupo et al., 2021b; Olivito et al., 2022a,b). All patients were diagnosed with bipolar disorder by an expert clinical psychiatrist from the Department of Psychiatry, Policlinico Umberto I Hospital using the Italian version of the Structured Clinical Interview for DSM-5 – Clinician Version (SCID-5-CV) (First et al., 2017). The following criteria were required for BD patient inclusion: (i) aged between 18 and 60 years, (ii) presence of euthymic mood for at least 6 months, (iii) first examination by a psychiatrist performed before age 40 years, (iv) suitability for magnetic resonance imaging (MRI), (v) absence of psychoactive substance use or alcohol abuse for at least 6 months, (vi) no comorbidity with other neurological or psychiatric disorders in Axis II-personality disorders, (vii) level of general intellectual functioning in the normal range, and (viii) absence of conditions, such as pregnancy, diabetes or cardiovascular disease. Moreover, to investigate the possible occurrence of motor disorders of cerebellar origin, a neurological assessment using the International Cooperative Ataxia Rating Scale (ICARS) (Trouillas et al., 1997) was performed, and the test results ranged from 0 (absence of motor deficit) to 100 (maximum severity of motor deficits). All patients underwent the ToM and sequential processing battery and the MRI protocols. The Hamilton Depression Rating Scale (HDRS score < 10) (Hamilton, 1967) and Young Mania Rating Scale (YMRS score < 12) (Young et al., 1978) were administered to confirm the euthymic phase. Moreover, all patients underwent a neuropsychological battery composed by Raven’s 47 Progressive Matrices test (Raven’s 47) (Raven, 1949), Wisconsin Card Sorting Test (Heaton, 1981), Tower of London Test (Krikorian et al., 1994), Trail Making Test, (Giovagnoli et al., 1996), Digit Forward Test (Orsini et al., 1987), and Corsi Forward Test (Corsi, 1972), to exclude the possible influence of intellectual functioning, executive functions, attention and working memory on patients’ performances. BD patients’ mean scores in the neuropsychological evaluation are reported in Supplementary Table 1. All patients were on medication at the time of assessment. The demographic and clinical characteristics and the scores obtained in the screening evaluation are reported in Table 1. Current pharmacotherapy of BD is reported in Supplementary Table 2.

**TABLE 1:** Demographic characteristics of BD patients and controls.

Group	N	F/M	Age	Education	Raven’s 47	ICARS	HDRS	YMRS
BD	18	12/6	39.00 (7.99)	15.56 (2.66)	27.64 (5.92)	1.08 (1.73)	1.67 (2.83)	0.94 (2.01)
HS	24	15/9	33.67 (11.06)	15.50 (2.87)	30.08 (2.02)	-	-	-

The data are reported as means and standard deviations. Raven’s 47: cut-off = < 18.96; BD = bipolar disorder; HS, healthy subjects; N, number; F, female; M, male; ICARS, International Cooperative Ataxia Rating Scale (Trouillas et al., 1997); HDRS, Hamilton Depression Rating Scale (Hamilton, 1967) YMRS, Young Mania Rating Scale (Young et al., 1978).

Two control groups were enrolled in the study. The first control group (HS) was recruited for ToM and sequential processing assessment and comprised 24 healthy subjects well-matched based on age (mean age/SD: 33.7/11.1), education (years of education/SD: 15.5/2.87), and gender distribution (M/F: 9/15) with no history of neurological or psychiatric illness. No significant difference was detected between the two groups in the mean age (*t* = −1.73; *p* = 0.09) or educational level (*t* = −0.0; *p* = 0.95) as evidenced by the *T* test analyses as well as gender distribution (χ2 = 0.08; *p* = 0.78) as assessed using the chi-squared (χ2) analysis. Raven’s 47 (Raven, 1949) was administered to HS and BD to assess intellectual level. The demographic characteristics and the scores obtained in the screening evaluation of HS are reported in Table 1.

The second control group (HS-MRI) included thirty-seven healthy subjects (mean age/SD: 45.76/14.26; M/F: 15/22) with no history of neurological or psychiatric diagnosis for whom MRI data were previously collected at the Neuroimaging Laboratory of Santa Lucia Foundation. Due to contraindications for the MRI procedure, one out of the 18 BD patients was excluded from the MRI protocol. *T* test analyses showed no significant difference in the mean age (*t* = 1.8; *p* = 0.75) or sex distribution (*t* = 0.13; *p* = 0.71) between the BD group (mean age/SD: 45.76/14.26; M/F: 6/11) and HS-MRI groups. This research study was conducted in accordance with the Declaration of Helsinki and approved by the Ethics Committee of Fondazione Santa Lucia (IRCCS). Informed written consent was obtained from all participants.

### 2.2. Behavioral assessment

The sequential processing and ToM battery was composed of two different tests.

The Sequence Test developed by Leggio and coworkers (Leggio et al., 2008) was specifically designed to differentiate sequential information processing by content. The test consists of 11 sets of cards, all of which need to be placed in a sequentially logical order. The cards represent three conditions: behavioral sequences represented by cartoon-like drawings and composed by 4 sets of cards, verbal sequences characterized by sentences and composed by 4 sets of cards, and spatial sequences composed by abstract figures and including 3 sets of cards. For non-verbal sequences, behavioral sequences assess the behavioral factor and need to be correctly sequenced by considering temporal, spatial and semantic information, whereas spatial figures evaluate the spatial factor and only require spatial clues to be properly sorted. The sentences analyze the verbal factor and are placed in sequence to form short stories with logical meaning. The scoring calculation was based on the different attribution given to the entire correct sequence compared to the score given for a single correctly sequenced fragment, and no time limits were imposed (see Leggio et al., 2008) for detailed information on the development, administration and scoring of the test; see Appendix in Supplementary material for examples of the sequences).

The Italian version of The Faux Pas test (FP) (Stone et al., 1998; Liverta Sempio et al., 2005) was used to assess the ability to make inferences about another person’s state of mind. This test investigates both cognitive (questions 1-5) and affective (question 6) components of ToM. The test includes 10 stories that comprise a social faux pas and 10 control stories with no social faux pas occurrence. All stories were read to subjects; subjects had a copy of the story to read along and check back over. For each of the 20 stories, the investigator asked subjects if anyone said something they should not have said. When a faux pas was identified, additional questions were asked to deepen the understanding of mental and emotional states. To correctly identify a social faux pas – that is, when a speaker says something without considering that the listener could not want to hear it or could be hurt by the statement – participants needed to understand that the person who committed the faux pas did not do so consciously and did not know that the interlocutor might feel upset by that. More specifically, they had to understand the inappropriateness of a behavior or action with respect to learned and predicted social norms. The events reported in the faux pas stories were unexpected and not univocal. The events required to be properly sequenced to consent a constant comparison between social expectations and the actual event, thus leading to a high prediction load. Conversely, in the no-faux pas stories, the social events were univocal and expected, requiring a low level of predictive ability. Every answer to faux pas stories’ questions was scored 1 if correct and 0 if wrong with a maximum score of 6. In the no-faux pas stories, a score of 2 was given if the participants correctly denied the presence of a faux pas. All stories included two control questions to verify whether the subjects understood the story. The questions were scored 1 if correct and 0 if wrong. The faux pas stories total score, the no-faux pas stories total score and the cognitive and affective components scores were considered for statistical comparisons (see Appendix in Supplementary material for examples of the stories).

### 2.3. Data analysis

For the present study, the sample size was calculated with *a priori* power analyses using G-power software and estimating the effect size from relevant data taken from a previous study (Olivito et al., 2022b). A total sample size of 42 individuals (18 BD patients and 24 HS) is required to obtain 95% power (alpha: 0.05) under the expectation of a large effect size (d = 1.09). In addition, the distribution of variables was verified using the Shapiro−Wilk test (Öztuna et al., 2006) which revealed that the scores did not exhibit a normal distribution across the BD and HS groups (p < 0.05). Therefore, the non-parametric Mann−Whitney U test for independent samples (p ≤ 0.05) was used to compare the performance between BD subjects and HS. The accuracy raw scores of each test were used for statistical comparison between groups. Spearman’s test correlational analysis was performed between the scores on the ToM and sequential tests and the scores obtained on the neuropsychological tests, the HDRS and the YMRS to exclude any possible influence of cognitive functions, depression scores and mania scores on behavioral results. Statistical analyses were performed using SPSS (Statistical Package for the Social Sciences, version 25).

### 2.4. MRI acquisition protocol

Patients and HS-MRI underwent MRI examination at 3T (Magnetom Allegra, Siemens, Erlangen, Germany) that included the following acquisitions: (1) dual-echo turbo spin echo [TSE] (TR = 6190 ms, TE = 12/109 ms); (2) fast-FLAIR (TR = 8170 ms, 204TE = 96 ms, TI = 2100 ms); and (3) 3D modified driven equilibrium Fourier transform (MDEFT) scans (TR = 1338 ms, TE = 2.4 ms, matrix = 256 × 224 × 176, in-plane FOV = 250 × 250 mm2, slice thickness = 1 mm) to perform voxel-based morphometry on cerebellar gray matter (GM) maps. To characterize the brain anatomy and determine the presence of macroscopic structural abnormalities, TSE scans of patients were visually inspected by an expert neuroradiologist. For HS-MRI, conventional MRI scans were reviewed to ensure the absence of any macroscopic brain abnormality.

### 2.5. MRI data processing and analysis

The individual preprocessing of the cerebellum was performed by using the Spatially Unbiased Infratentorial Template (SUIT) toolbox (Diedrichsen et al., 2009) implemented in Statistical Parametric Mapping version 8 (Wellcome Department of Imaging Neuroscience; SPM-8, accessed on 2 April 2009)^1^. The procedure included the following processing steps for each participant’s individual T1 anatomical images: the cerebellum was isolated, the isolated maps were hand-corrected if necessary, and each cropped image was normalized into SUIT space; the deformation parameters obtained by normalization were used to reslice the probabilistic cerebellar atlas into individual subjects’ space, and the images were smoothed using an 8-mm FWHM Gaussian kernel.

Voxel-based morphometry was executed on cerebellar modulated GM maps entered into a voxelwise two-sample t test model as implemented in SPM-8 to distinctly compare the cerebellar GM volumes between the BD group and HS-MRI group. Sex and age were entered as variables of no interest, and the analysis was restricted to the voxels of the cerebellum by using an explicit exclusion mask. The results were considered significant at *p* values < 0.05 FWE corrected at the cluster level.

## 3. Results

### 3.1. Behavioral profile

**FIGURE 1:**
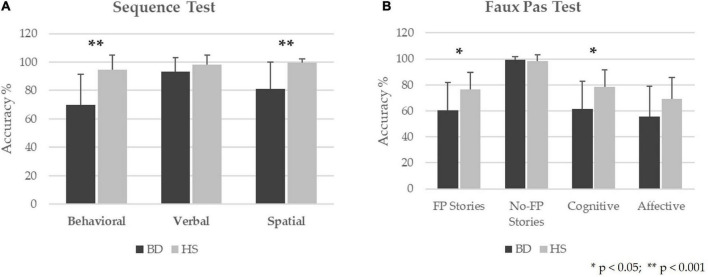
Behavioral results in BD patients as compared to HS. The results for Sequence Test **(A)** and Faux Pas Test **(B)** are presented as the percentage of the total number of correct responses (accuracy); 0% indicates no correct answers, and 100% indicates totally correct answers. The mean and standard deviation of the accuracy are reported for BD and HS. ** Statistical significance at *p*-values < 0.001. *Statistical significance at *p*-values < 0.05.

In the Sequence Test, BD patients showed significant differences as compared to HS in the behavioral sequences (Mann-Whitney U - MWU = 78; *Z* = −3.65; *p* = 0.00006), the spatial sequences (MWU = 97.5; *Z* = −3.69; *p* = 0.00007), whereas a trend toward a significant difference was detected in the verbal sequences (MWU = 156; *Z* = −1.93; *p* = 0.053). The percentages of accuracy are reported in Figure 1A.

Regarding the Faux Pas test, significant differences between the two groups were found in the total score of the “faux pas” stories (MWU = 130.5; *Z* = −2.17; *p* = 0.03) and the cognitive component scores (MWU = 130; *Z* = −2.19; *p* = 0.029). In contrast, no significant differences were found in the total score of the “no-faux pas” stories (MWU = 211; *Z* = −0.22; *p* = 0.82) and the affective component scores (MWU = 149; *Z* = −1.72; *p* = 0.08). The percentages of accuracy are calculated as the percentage of correct responses in each test and are reported in Figure 1B. Descriptive statistics and results of statistical analyses performed by Mann-Whitney U for each test are reported in Table 2.

**TABLE 2:** Descriptive statistics and results of statistical analysis between BD and HS in the Sequence and Faux Pas test results.

Group	Sequence test			Faux pas test			
	*Behavioral*	*Verbal*	*Spatial*	*FP Stories*	*No-FP Stories*	*Cognitive*	*Affective*
BD	2.78, 3 (0.86)	3.73, 4 (0.39)	2.43, 2.6 (0.57)	36.27, 35.5 (12.81)	19.83, 20 (0.51)	30.72, 30 (10.8)	5.55, 5.5 (2.33)
HS	3.79 4 (0.40)	3.92 4 (0.26)	2.98 3 (0.08)	44.83 44.5 (9.57)	19.66 20 (0.96)	38.12 39 (8.2)	6.7 7 (1.92)
BD vs HS	0.00006**	0.053	0.00007**	0.030*	0.821	0.029*	0.085

The data are reported as means, medians and standard deviations for descriptive statistics and as p-values for statistical analyses performed by Mann-Whitney U test for independent samples. BD, bipolar disorder; HS, healthy subjects. **Results significant at *p* < 0.001. *Results significant at *p* < 0.05.

Spearman’s test correlational analysis revealed a significant and meaningful correlation between the behavioral condition of the sequence test and scores in the Corsi forward test. No substantial correlations were found between ToM and the other sequential tasks as well as the scores obtained for the other neuropsychological tests, the HDRS and the YMRS. For detailed statistics, see Supplementary Table 3 in the Supplementary materials.

### 3.2. Cerebellar voxel-based morphometry

**FIGURE 2:**
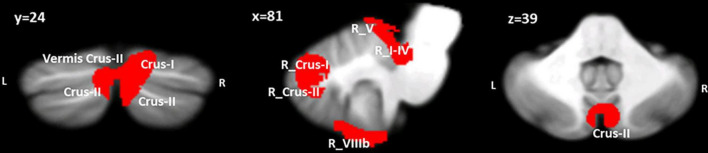
Between-group voxel-based comparison of cerebellar GM density. Clusters of significantly reduced cerebellar GM in BD compared to HS-MRI are reported and superimposed on the Spatially Unbiased Infratentorial Template (SUIT) (Diedrichsen et al., 2009) in coronal (y), sagittal (x) and axial (z) slices. The results are significant at *p*-values < 0.05 after FWE cluster-level correction. L: left; R: right.

The VBM results evidenced the occurrence of structural cerebellar alterations in BD patients with respect to the second control group (HS-MRI) enrolled for the MRI experiment. The patterns of reduced GM density in BD patients involved both the anterior and posterior cerebellar regions. Two clusters were identified. A larger cluster of significant GM reduction was found with peak voxels in the right Crus II, whereas the second cluster had peak voxels in right lobules I-IV and V with extensions to the right lobule VI, Crus I, VIIIA, VIIIB, IX, left Crus I and II, vermis Crus II, vermis VI, and vermis VIIIA (Figure 2). Detailed statistics and peak voxels showing the greatest significant differences in a cluster are reported in Table 3.

**TABLE 3:** Detailed statistics of voxel wise comparisons of cerebellar GM density (BD < HS-MRI).

Cluster size (NoV)	Coordinates			Peak z-score	Cerebellar region
	x	Y	z		
8,296	4	−78	−35	4.80	Right Crus II
2,889	8	−35	−18	5.63	Right Lobule I-IV
	18	−37	−15	5.25	Right Lobule V

^1^Results are significant at *p* < 0.05 after FWE correction.

## 4. Discussion

The key role of the cerebellum in adaptive control and predictive coding by means of a sequential operational mode has been documented by many studies in diverse domains ranging from sensorimotor to advanced cognitive and ToM functions (Ito, 2008; Molinari et al., 2008; Leggio et al., 2011; D’Angelo and Casali, 2012; Sokolov et al., 2017; Clausi et al., 2019; Heleven et al., 2019; van Overwalle et al., 2019a,b). Nevertheless, thus far, most of those studies have focused on investigating the cerebellar operational mode in healthy individuals and in patients with pure cerebellar lesions or degenerative disorders. Cerebellar alterations and ToM deficits have been described in patients with remitted BD (Shamay-Tsoory et al., 2009; Bora et al., 2016; Argyropoulos et al., 2021; Olivito et al., 2022b). However, no study has previously focused on the ability to sequence and predict events in this clinical population. To address this gap in the current study, we investigated the performance of individuals with bipolar disorder in the euthymic phase in non-explicitly sequential ToM tasks and sequential non-ToM tasks, both requiring prediction capacities. In addition, VBM analysis was used to explore the patterns of cerebellar GM and assess whether our sample of BD patients showed the concomitant presence of cerebellar structural alterations and sequential and predicting deficits.

BD patients performed significantly worse in the Faux Pas Test than HS. Specifically, the ability to identify the occurrence of the faux pas and to justify others’ behavior when the faux pas was correctly identified were impaired in BD patients, whereas a trend toward significantly worse performance was detected for the affective component. Our results on preserved affective component of ToM are in line with previous studies on BD (Montag et al., 2010; Mercer and Becerra, 2013). While most studies have regularly reported deficits in the cognitive component of ToM in BD, inconsistencies have often occurred as concerned with the affective component (Shamay-Tsoory et al., 2009; Ioannidi et al., 2011). In the present study, BD revealed to be able to “feel” the emotional impact that an inconvenient situation could have on the person who might be hurt by it, but they were unable to use this information to provide appropriate justifications on the protagonist’s behavior, demonstrating cognitive ToM difficulties. In previous studies it has been speculated that BD patients typically generate extreme emotional responses due to an over activation of limbic regions during the appraisal of emotional material (Shamay-Tsoory et al., 2009). This hyperactivation has also been hypothesized to account for “hyper empathy” that BD show and to be related to affective ToM inconsistency found among studies, in which BD often reveal to process affective ToM information as healthy subjects do (Shamay-Tsoory et al., 2009). Additionally, affective ToM impairments are reported to be higher during the acute phases of BD, and to normalize during the euthymic phases (Bora et al., 2016). This effect might be related to affective regulation and stabilization due to medications, that might impact more on affective processing of stimuli rather than on cognitive appraisal of those. The ability to adequately respond to the questions posed by the examiner partly entails sequential processing; however, it is not as explicitly required as noted in the Sequence Test. Indeed, to identify the faux pas and to understand that a person has said something awkward with respect to expected behavior, it is necessary for the individual to implicitly understand the correct sequence of social actions occurring in the story and to detect an error compared to a properly predicted behavior (Clausi et al., 2019). In stories with no faux pas, BD patients exhibited performance equal to HS. Indeed, in these stories, it is easier to predict others’ social behavior given that the social situations present no errors to be detected. Thus, when no conflict occurs between expected and current conditions, individuals can rely on internal models of previous experiences with no need for correction, whereas a higher predictive ability is crucial to properly identify the faux pas occurrence. In the latter, questions are asked about why the person committed a faux pas and why he or she should not have done so. Thus, this test investigates a complex level of ToM that entails the ability to predict and verbalize inferences about specific consequences of a series of unexpected and inconvenient events, requiring online comparisons between external stimuli and internalized models of social expectations (de Siqueira Rotenberg et al., 2020).

An explicit demand in terms of sequential processing is associated with the Sequence Test, whereas no ToM component is involved. In this test, BD patients exhibited poorer performance than HS in all three conditions examined, although for the verbal sequences the difference only denotes a trend toward a significance. Our results revealed that sequential difficulties are experienced by these patients despite the content of the stimuli. In the Sequence Test, the high sequential demand is associated with the need to detect the errors associated with the non-logical sequence as presented to the subjects, to identify the proper associations between events, to determine precedence and to finally place events in chronological order to logically reconstruct the sequence (Leggio et al., 2008). The requisite to assemble cards in the proper order implies making predictive connections among the cards. Consequently, the sequence misinterpretation might be due to the inability to detect the errors in the non-logical sequences presented and thus to properly predict the way each card is temporarily and spatially linked to the next. Previous studies found that patients with cerebellar disease failed to accomplish this test regardless of the specific content (Leggio et al., 2008; Tedesco et al., 2011).

The pattern of GM reduction found in BD patients includes both anterior and posterior cerebellar lobules, which is consistent with the results of previous studies we performed using BD patients (Lupo et al., 2021b; Olivito et al., 2022b). The reduced GM in the anterior cerebellar areas might be related to psychomotor agitation that typically characterizes affective episodes (Kraepein, 1921; Sani et al., 2016) and that could lead to persisting structural changes in motor and sensorimotor regions of the cerebellum, such as lobules I-IV and V (Lupo et al., 2021b). On the other hand, the GM reduction in the posterior cerebellum is consistent with the behavioral profile of BD patients and with the well-known functional topography of the cerebellum (Sokolov, 2018; Stoodley and Schmahmann, 2018). Indeed, a larger cluster of significant GM reduction was evidenced in the right Crus II, a region known to be involved in mentalizing processes, primarily when a high level of abstraction is in demand (van Overwalle and Mariën, 2016; van Overwalle et al., 2020). Several fMRI studies on healthy subjects have acknowledged the activation of the posterior cerebellum, specifically the Crus I and II, together with cerebral areas known to be part of the social brain during social mentalizing both when the tasks required and did not require sequential processing (van Overwalle and Mariën, 2016; van Overwalle et al., 2022). Together with functional activation, cerebellar Crus II and mentalizing brain regions have been found to be effectively connected by closed loops, further demonstrating the cerebellar modulation of advanced social processing (van Overwalle et al., 2020). In addition, dysfunctional advanced ToM skills, which were investigated by means of the FP test, have been previously related to decreased GM in cerebellar Crus II in patients with pure cerebellar disease (Clausi et al., 2019, 2021a,b) and in individuals with autism spectrum disorders (Clausi et al., 2021b) and bipolar disorder types 1 and 2 (Olivito et al., 2022b). Coherently, altered functional connectivity between the Crus II and social mentalizing areas has been reported in all the above-mentioned clinical conditions (Olivito et al., 2018, 2022a; Clausi et al., 2019). The most sustained hypothesis holds that structural alterations in the cerebellar posterior areas might impede the proper functional modulation that the cerebellum exerts on regions of the neocortex, thus affecting appropriate high-order functions (Clausi et al., 2019). Additionally, the interplay among the posterior cerebellum, the prefrontal cortex and temporoparietal cortex is associated with the ability of the human brain to construct internal models of complex stimuli occurring in the external world and to control inner manipulation of related processing and thought (Ito, 2008). Consistently, a complex human process such as prediction error, recruits the most lateral part of the cerebellum (Ploghaus et al., 2000). Additionally, activity in the right posterior cerebellum is triggered when people are asked to predict an event in the future, whereas no cerebellar activation was detected for past episode recollection. These findings likely indicate that the posterior cerebellum might be pivotal for the creation or reshaping of novel internal models based on experiential models (Szpunar et al., 2006; Ito, 2008).

The prediction capacity of the cerebellum is currently considered a supramodal function that modulates whole-brain function alerting specialized brain circuits based on the type of incoming input and of the response required in the specific context (Tesche and Karhu, 2000; Restuccia et al., 2007; Moberget et al., 2014; Molinari and Masciullo, 2019). The participation of the cerebellum in the predictive brain is ascribed to its role in detecting sequences and violations from correct sequences and creating internal models of those sequences used to make proper predictions, thus allowing feedforward control based on anticipation (Molinari and Masciullo, 2019). Sequential processing represents the basic functional mechanism of the cerebellum in several functional domains, including motor and cognitive control, language, spatial processing, decision-making, and theory of mind (Braitenberg et al., 1997; Leggio et al., 2008; Molinari et al., 2008; Tedesco et al., 2011; Clausi et al., 2019; van Overwalle et al., 2019b). As previously stated, poor performance in the Sequence Test has been reported in patients with cerebellar diseases (Leggio et al., 2008; Tedesco et al., 2011). Specifically, patients with lesions occurring in the posterior cerebellum were associated with worse performances (Tedesco et al., 2011). Interestingly, our sample of BD patients showed altered performances in the Sequence Test in presence of a large cluster of decreased cerebellar GM in posterior regions, such as the right lobule Crus II with extensions in the right lobules VI and Crus I and in the left Crus I-II.

It is worth noting that no influences of intellectual level and executive functions were found on patients’ sequences and ToM performances, as revealed by the absence of correlations among those (see Supplementary Table 3 in Supplementary materials). The only correlation we found occurred between the behavioral condition of the Sequence Test and the Corsi forward test. This result is consistent with the knowledge that to recognize the proper order of specific stimuli, the information extrapolated from one stimulus and the comparisons and the predictive link between stimuli must be kept active in a working memory system (Leggio et al., 2008, 2011).

Overall, the results of the present study indicate BD patients’ impairment in ToM and sequential abilities that require prediction processing and error detection and correction to accomplish the requested tasks. Consistent with widespread literature, we suggest that the above-mentioned compromised performances might be related to reduced GM occurring in the posterior cerebellar areas. It is likely that the structural alterations in the posterolateral cerebellum might affect the cerebellar modulation on cortical projection areas, which is necessary to inform about the proper sequential responses to be made when unexpected events are met. As a supramodal function of the cerebellum, prediction failure might influence cognitive and social domains in various components, thus impeding the optimization of a range of functions and behaviors (Clausi et al., 2019). Accordingly, damage to the cerebellum has been described as a shared risk factor among different psychopathologies, emphasizing the usefulness of focusing on transdiagnostic dimensional features between categorical mental disorders for both empirical research and applied practice (Romer et al., 2018; Siciliano et al., 2022). In this regard, we suggest that the impact of prediction deficits should be considered when assessing a patient’s psychosocial functioning and his or her quality of life. In the context of our study, we propose that the imbalanced mood homeostasis typical of BD might be accompanied by an impaired capacity to properly process temporal and spatial information between stimuli, occurring in both the external and internal world. Under normal conditions, the cerebellum is needed to create appropriate internal models of actual events for use in the future for proper interpretations and prediction of events and, in general, to adapt behaviors to a given context and situation. Thus, our preliminary results provide the breeding ground for future hypothesis-testing studies assessing the causal link between cerebellar alterations and sequential and predictive deficits in BD.

Some limitations of the present study must be mentioned. Our sample of BD patients included both patients with BD types 1 and 2 given that the total number of patients recruited did not allow us to differentiate and characterize the two different subtypes. Thus, future research is needed to further characterize predictive ToM and sequential processing and the direct link with subtending cerebellar structural alterations in the two BD subtypes.

Finally, as reported in Supplementary Table 2, the BD patients enrolled were medicated, many of whom were receiving treatment involving polypharmacotherapy. Indeed, the enrollment of drug-free patients was not possible due to ethical and clinical concerns and given that pharmacotherapy is essential for patients to achieve a clinical stability pivotal for remission of symptoms and to maintain a euthymic state. In addition, it has to be considered that, although it is possible that research with participants receiving psychotropic medications might reveal confounding imaging results, most of the evidence from morphometric imaging studies shows that the effect of medication is unlikely to explain the neuroimaging parameters’ variances occurring between bipolar individuals and healthy controls (Hafeman et al., 2012).

Additionally, the present study does not provide evidence of a causal link between the observed changes in GM in the cerebellum and the reported performances on behavioral tasks. In particular, since we did not detect a monotonic relationship between each pair of variables, namely between values of GM reduction in each lobule and the performances at each SC tests, we could not conduct correlation analysis.

Despite these limitations and the preliminary nature of the research, the present study represents the first investigation proving the presence of sequencing and predicting deficits in BD patients, and the first step to provide a theoretical link between these deficits and cerebellar alterations.

## Data availability statement

The raw data supporting the conclusions of this article will be made available by the authors, without undue reservation.

## Ethics statement

The studies involving human participants were reviewed and approved by Ethics Committee of Fondazione Santa Lucia IRCCS (Prot. CE/PROG.579 and 22 November 2016). The patients/participants provided their written informed consent to participate in this study.

## Author contributions

LS, GO, MLu, and MLe: conceptualization. LS, GO, and MLu: methodology, writing—original draft preparation, and writing—review and editing. LS, GO, MLu, and NU: formal analysis and data curation. LS, GO, MLu, NU, AG, and MS: investigation. RD and MLe: supervision. All authors have read and agreed to the published version of the manuscript.

## Footnotes

1
https://www.fil.ion.ucl.ac.uk/spm/

## Appendix

**Sequence Test**

Set of cards representative of the three conditions and correctly sequenced. As follows the English translation of the “verbal sequence” example: Luciano came back from school/and his mother fixed him lunch;/he did his homework/and ate a snack,/after he went out to play with friends/and he came back for dinner.

**Faux Pas Test**

Example of ‘Faux Pas story’: Helen’s husband was throwing a surprise party for her birthday. He invited Sarah, a friend of Helen’s, and said, “Don’t tell anyone, especially Helen.” The day before the party, Helen was over at Sarah’s and Sarah spilled some coffee on a new dress that was hanging over her chair. “Oh!” said Sarah, “I was going to wear this to your party!” “What party?” said Helen. “Come on,” said Sarah, “Let’s go see if we can get the stain out.”

1. Did anyone say something they shouldn’t have said or something awkward?
If yes, ask:
2. Who said something they shouldn’t have said or something awkward?
3. Why shouldn’t he/she have said it or why was it awkward?
4. Why do you think he/she said it?
5. Did Sarah remember that the party was a surprise party?
6. How do you think Helen felt?

Control question:

1. In the story, who was the surprise party for?
2. What got spilled on the dress?

Example of ‘No Faux Pas story’: Vicky was at a party at her friend Oliver’s house. She was talking to Oliver when another woman came up to them. She was one of Oliver’s neighbours. The woman said, “Hello,” then turned to Vicky and said, “I don’t think we’ve met. I’m Maria, what’s your name?” “I’m Vicky.” “Would anyone like something to drink?” Oliver asked.

1. Did anyone say something they shouldn’t have said or something awkward?
If yes, ask:
2. Who said something they shouldn’t have said or something awkward?
3. Why shouldn’t he/she have said it or why was it awkward?
4. Why do you think he/she said it?
5. Did Vicky and Maria know each other?
6. How do you think Vicky felt?

Control questions:

1. In the story, where was Vicky?
2. Who was hosting the party?
