# Supplementary material for: The role of the cerebellum in sequencing and predicting social and non-social events in patients with bipolar disorder
Source: Front Cell Neurosci. 2023 Feb 15;17:1095157. doi: 10.3389/fncel.2023.1095157 (PMC9974833; doi:10.3389/fncel.2023.1095157)
Supplement: Supplementary file 1 [file Data_Sheet_1.PDF]

Supplementary Material

Table S1. Neuropsychological Tests' Mean Scores in BD population and cut-off in Normative Data

	Trail A	Trail B	Trail A-B	ToL	WCST - Errors	Digit F Span	Corsi F Span
**BD**	48.31 (9.65)	107.47 (30.60)	59.84 (30.43)	29.72* (3.03)	108.56* (15.93)	6.06** (1.26)	4.94 (0.87)
**Cut-off**	> 93	> 282	> 186	27-36	< 85	< 5	< 3

The patients' performance to each test was considered impaired when the score was below the cut-off value, with exception of the Trail-making test (Trial A, Trial B, Trial A-B) in which the performance resulted impaired when the scores were higher than the cut-off values. * 2 patients had scores under the cut-off normative data. ** 3 patients had scores under the cut-off normative data.

Table S2. Pharmacological treatment in BD patients.

N	Pharmacotherapy
15	Antiepileptics
9	Antipsychotics
8	Lithium
2	Anxiolytics
1	Antidepressants
11	Polypharmacy

N = Number of BD patients taking the medication. Polypharmacy = Patients taking at least two different types of medication.

Table S3. Correlations between Faux Pas Test and Sequencing Test scores and Neuropsychological Tests.

	Sequence Test			Faux Pas Test			
	BE	*VE*	*SP*	*FP stories*	*No-FP Stories*	*Cognitive*	*Affective*
**HDRS**	R = 0.01<br>p = 0.97	R = -0.13<br>p = 0.60	R = -0.20<br>p = 0.42	R = 0.14<br>p = 0.59	R = -0.45<br>p = 0.06	R = 0.22<br>p = 0.38	R = -0.04<br>p = 0.89
**YMRS**	R = 0.18<br>p = 0.47	R = -0.18<br>p = .047	R = -0.23<br>p = 0.36	R = 0.22<br>p = 0.38	R = -0.28<br>p = 0.27	R = 0.19<br>p = 0.45	R = 0.32<br>p = 0.20
**Trail A**	R = 0.11<br>p = 0.66	R = 0.00<br>p = 0.99	R = 0.22<br>p = 0.38	R = 0.04<br>p = 0.88	R = 0.08<br>p =0.77	R = 0.03<br>p = 0.90	R = 0.01<br>p = 0.98
**Trail B**	R = -0.07<br>p = 0.79	R = 0.65<br>p = 0.00	R = 0.17<br>p = 0.51	R = -0.44<br>p = 0.07	R = 0.33<br>p = 0.19	R = -0.39<br>p = 0.11	R = -0.38<br>p = -12
**Trail A-B**	R = -0.15<br>p = 0.56	R = 0.71<br>p = 0.00	R = 0.04<br>p = 0.87	R = -0.37<br>p = 0.13	R = 0.40<br>p = 0.10	R = -0.33<br>p = 0.18	R = -0.29<br>p = 0.24
**ToL**	R = 0.35<br>p = 0.16	R = -0.24<br>p = 0.34	R = 0.42<br>p = 0.08	R = -0.04<br>p = 0.87	R = -0.16<br>p = 0.53	R = -0.06<br>p = 0.81	R = -0.06<br>p = 0.82
**WCST - Errors**	R = 0.25<br>p = 0.31	R = 0.46<br>p = 0.05	R = 0.25<br>p = 0.31	R = 0.06<br>p = 0.81	R = 0.43<br>p = 0.07	R = 0.10<br>p = 0.70	R = -0.04<br>p = 0.86
**Digit F Span**	R = -0.03<br>p = 0.90	R = 0.18<br>p = 0.46	R = 0.19<br>p = 0.44	R = 0.15<br>p = 0.55	R = 0.31<br>p = 0.21	R = 0.11<br>p = 0.67	R = 0.32<br>p = 0.20
**Corsi F Span**	***R = 0.65***<br>***p = 0.00***	R = -0.13<br>p = 0.60	R = 0.24<br>p = 0.34	R = 0.22<br>p = 0.38	R = 0.20<br>p = 0.44	R = 0.22<br>p = 0.37	R = 0.15<br>p = 0.54
**Raven's 47**	R = 0.41<br>p = 0.09	R = -0.13<br>p = 0.60	R = 0.10<br>p = 0.70	R = 0.24<br>p = 0.34	R = -0.29<br>p = 0.24	R = -0.19<br>p = 0.46	R = -0.42<br>p = 0.08

Correlations significant at p=<0.05 are presented in bold and italic type. BE = Behavioral; VE = Verbal; SP = Spatial; Digit F = Digit Forward; Corsi F = Corsi forward; HDRS = Hamilton Depression Rating Scale; YMRS = Young Mania Rating Scale. Attention was measured by Trail-making test (Trail A, Trail B, Trail A-B). Executive functions were measured by Tower of London Test (ToL) and Wisconsin Card Sorting Test (WCST). Working memory was measured by Digit F Span and Corsi F Span. Intellectual functioning was measured by Raven's 47.

Appendix

Sequence Test

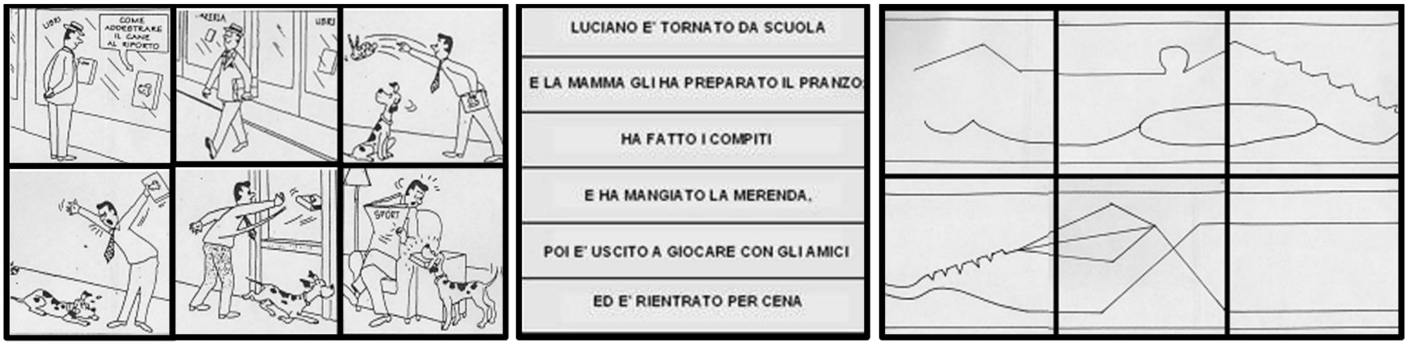

Behavioral Sequence Verbal Sequence Spatial Sequence

Set of cards representative of the three conditions and correctly sequenced. As follows the English translation of the “verbal sequence” example: Luciano came back from school/ and his mother fixed him lunch;/ he did his homework/ and ate a snack, / after he went out to play with friends /and he came back for dinner.

Faux Pas Test

Example of ‘Faux Pas story’: Helen's husband was throwing a surprise party for her birthday. He invited Sarah, a friend of Helen's, and said, "Don't tell anyone, especially Helen." The day before the party, Helen was over at Sarah's and Sarah spilled some coffee on a new dress that was hanging over her chair. "Oh!" said Sarah, "I was going to wear this to your party!" "What party?" said Helen. "Come on," said Sarah, "Let's go see if we can get the stain out."

1. Did anyone say something they shouldn't have said or something awkward?

If yes, ask:

2. Who said something they shouldn't have said or something awkward?
3. Why shouldn't he/she have said it or why was it awkward?
4. Why do you think he/she said it?
5. Did Sarah remember that the party was a surprise party?
6. How do you think Helen felt?

Control question:

1. In the story, who was the surprise party for?
2. What got spilled on the dress?

Supplementary Material

Example of ‘No Faux Pas story’: Vicky was at a party at her friend Oliver’s house. She was talking to Oliver when another woman came up to them. She was one of Oliver’s neighbours. The woman said, "Hello," then turned to Vicky and said, " I don't think we've met. I’m Maria, what's your name?" "I’m Vicky." "Would anyone like something to drink?" Oliver asked.

1. Did anyone say something they shouldn't have said or something awkward?

If yes, ask:

2. Who said something they shouldn't have said or something awkward?
3. Why shouldn't he/she have said it or why was it awkward?
4. Why do you think he/she said it?
5. Did Vicky and Maria know each other?
6. How do you think Vicky felt?

Control questions:

1. In the story, where was Vicky?
2. Who was hosting the party?
